# Sex associations and computed tomography coronary angiography-guided management in patients with stable chest pain

**DOI:** 10.1093/eurheartj/ehz903

**Published:** 2019-12-28

**Authors:** Kenneth Mangion, Philip D Adamson, Michelle C Williams, Amanda Hunter, Tania Pawade, Anoop S V Shah, Stephanie Lewis, Nicholas A Boon, Marcus Flather, John Forbes, Scott McLean, Giles Roditi, Edwin J R van Beek, Adam D Timmis, David E Newby, David A McAllister, Colin Berry

**Affiliations:** 1 British Heart Foundation Glasgow Cardiovascular Research Centre, Institute of Cardiovascular and Medical Sciences, University of Glasgow, 126 University Place, University of Glasgow, Glasgow G12 8TA, UK; 2 British Heart Foundation Centre for Cardiovascular Science, University of Edinburgh, 49 Little France Crescent, Edinburgh EH16 4SA, UK; 3 Christchurch Heart Institute, University of Otago, 2 Riccarton Avenue, Christchurch Central City, Christchurch 8011, New Zealand; 4 Institute of Health and Wellbeing, University of Glasgow, University Ave, Glasgow G12 8QQ, UK; 5 Centre for Population Health Sciences, University of Edinburgh, Teviot Place, Edinburgh EH8 9AG, UK; 6 Norwich Medical School, University of East Anglia, Norwich, NR4 7TJ, UK; 7 Graduate Entry School, University of Limerick, Limerick, St Nessan's Rd, Dooradoyle, Co. Limerick, V94 F858, Ireland; 8 National Health Service, Hayfield House, Hayfield Rd, Kirkcaldy KY2 5AH, Fife, UK; 9 William Harvey Research Institute, Queen Mary University of London, London EC1M 6BQ, UK

**Keywords:** Coronary heart disease, Angina, Gender, CTCA, CT coronary angiography

## Abstract

**Aims:**

The relative benefits of computed tomography coronary angiography (CTCA)-guided management in women and men with suspected angina due to coronary heart disease (CHD) are uncertain.

**Methods and results:**

In this *post hoc* analysis of an open-label parallel-group multicentre trial, we recruited 4146 patients referred for assessment of suspected angina from 12 cardiology clinics across the UK. We randomly assigned (1:1) participants to standard care alone or standard care plus CTCA. Fewer women had typical chest pain symptoms (*n* = 582, 32.0%) when compared with men (*n* = 880, 37.9%; *P* < 0.001). Amongst the CTCA-guided group, more women had normal coronary arteries [386 (49.6%) vs. 263 (26.2%)] and less obstructive CHD [105 (11.5%) vs. 347 (29.8%)]. A CTCA-guided strategy resulted in more women than men being reclassified as not having CHD {19.2% vs. 13.1%; absolute risk difference, 5.7 [95% confidence interval (CI): 2.7–8.7, *P* < 0.001]} or having angina due to CHD [15.0% vs. 9.0%; absolute risk difference, 5.6 (2.3–8.9, *P* = 0.001)]. After a median of 4.8 years follow-up, CTCA-guided management was associated with similar reductions in the risk of CHD death or non-fatal myocardial infarction in women [hazard ratio (HR) 0.50, 95% CI 0.24–1.04], and men (HR 0.63, 95% CI 0.42–0.95; *P*_interaction_ = 0.572).

**Conclusion:**

Following the addition of CTCA, women were more likely to be found to have normal coronary arteries than men. This led to more women being reclassified as not having CHD, resulting in more downstream tests and treatments being cancelled. There were similar prognostic benefits of CTCA for women and men.

## Introduction

In the management of suspected stable angina, women are less likely than men to be referred for cardiac investigations or undergo coronary revascularization.^1^ This is despite a higher prevalence of angina^2^ and a 50% higher lifetime risk of dying from coronary heart disease (CHD).^3^ Differences in clinical presentation in women contribute to under-recognition and less intensive treatment,^4^^,^^5^ and research studies in CHD may represent women less.^6^

Patients with stable chest pain are evaluated using anatomical imaging with computed tomography coronary angiography (CTCA) or functional testing including stress electrocardiography, radionuclide scintigraphy, echocardiography, or magnetic resonance imaging.^7–11^ In the Scottish Computed Tomography of the Heart (SCOT-HEART) trial, we reported that among patients referred for the evaluation of stable chest pain, CTCA clarified the diagnosis and altered subsequent management.^12^ At 5 years, CTCA-guided management added to standard care reduced the rate of death from CHD or non-fatal myocardial infarction (MI).^13^

We investigated whether treatment and outcomes following CTCA-guided management differ between women and men. We hypothesized that there are sex differences for the diagnosis of CHD, patient management (including investigations and treatment), and clinical outcomes, including CHD death and MI, at 5 years.

## Methods

### Study population

The SCOT-HEART study was a prospective clinical trial investigating the role of CTCA in patients aged between 18 and 75 years, referred to a cardiology clinic with suspected angina due to CHD. Patients with a prior history of CHD were eligible to participate. The standard care clinical assessment included exercise electrocardiography. The study design and principal findings^12^^,^^13^ have been reported previously. The study population was randomized 1:1 to standard care or standard care plus ≥64-slice CTCA using a web-based system. Patients gave written informed consent.

### Procedures

Cardiovascular risk was calculated with the ASSIGN score. ASSIGN has been developed, calibrated and validated for use in the UK.^14^

Obstructive coronary artery disease was defined as a luminal stenosis >70% in one or more major epicardial vessel, or >50% stenosis in the left main stem.^15^

At 6 weeks, attending clinicians were asked to review patients' diagnosis and management in view of all available information including the CTCA report (standard care plus CTCA) or the ASSIGN score (standard care alone). The clinician documented changes in diagnosis, investigations (stress testing or invasive coronary angiography), or treatments (preventive and antianginal treatments). Anginal symptoms were assessed by a self-administered Seattle Angina Questionnaire^16^ with telephone follow-up for non-responders after two mailings 2 weeks apart.

### Outcomes

The primary outcome of the trial was the proportion of patients diagnosed with angina secondary to CHD at 6 weeks. A false-positive or negative baseline diagnosis was determined to have occurred when the treating clinician changed the diagnosis at 6 weeks. Key secondary outcomes included changes in treatment or investigations at 6 weeks; CTCA findings; and changes from baseline in Seattle Angina Questionnaire after 6 weeks and 6 months.^12^ The principal clinical endpoints included the composite of death due to CHD or non-fatal MI and coronary revascularization procedures. These events were identified with data from the Information and Statistics Division of the National Health Service (NHS) Scotland and, when appropriate, confirmed by review of patient health records.^13^

### Statistical analyses

We performed a *post hoc* analysis stratified by sex. The analyses were performed according to the intention-to-treat principle. Missing data were removed from the analyses, except for data on deaths, which were censored at the time the patient was lost from the trial.

The diagnoses of CHD and angina due to CHD were assessed for certainty (yes/no vs. unlikely/probable in the primary analysis) and frequency (yes/probable vs. unlikely/no) of diagnoses.

Changes in diagnosis, planned investigations, and medical therapies were analysed within mixed-effects logistic regression models to calculate odds ratio with sex included as an interaction term. We obtained standard errors for absolute risk reduction for each sex assuming that the difference in risk between CTCA and control arm was approximately normal. The standard error for difference in absolute risk reduction between men and women was estimated as the square root of the sum of the standard errors squared for each sex. As some of the numbers were small, we repeated this analysis using simulation (sampling from Beta distributions) obtaining very similar results. Results are reported as odds ratios and absolute risk reductions with 95% confidence intervals (CIs). Clinical endpoint events were analysed with Cox regression models, similarly adjusted, and cumulative event curves were constructed.

All analyses were performed using R software, version 3.5.0 (R Foundation for Statistical Computing). Anonymized data will be made available on request.

## Results

### Characteristics of the study participants

Between 18 November 2010 and 24 September 2014, 4146 (42%) of 9849 patients who had been referred for assessment of suspected angina at 12 cardiology centres across the UK were enrolled and randomly assigned to standard care or standard care and CTCA.

**Figure 1:**
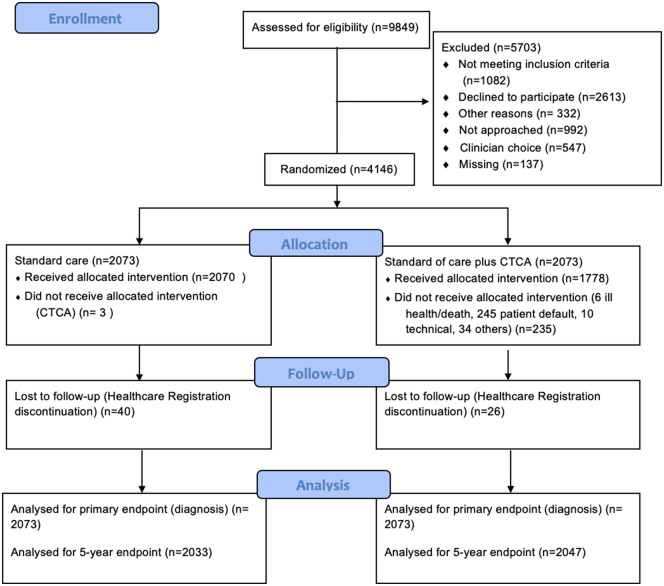
Trial design

Of 4146 randomized patients, 1821 (44%) were women (*Table 1* and *Figure 1*). Demographics and comorbidities were evenly distributed between the randomized groups. Compared to men, women had a lower frequency of prior CHD in the standard care [49 (5.4%) vs. 137 (11.8%), *P* < 0.001] and CTCA-guided groups [49 (5.4%) vs. 137 (11.8%), *P* < 0.001]. Fewer women were classified as having ‘typical’ chest pain symptoms in the CTCA-guided group [women: 281 (30.9%) vs. men: 456 (39.2%); *P* < 0.001] although no difference was demonstrated within the standard care group [women: 301 (33.1%) vs. men: 424 (36.5%); *P* = 0.117]. Women were half as likely to be referred for invasive angiography in both groups [standard care, women 76 (8.4%) vs. men 184 (15.8%); CTCA, women 59 (6.5%) vs. men 196 (16.9%)].

**Table 1:** Characteristics of the participants prior to randomization according to sex

	Standard care		*P*-value (women vs. men)	Standard care + CTCA		*P*-value (women vs. men)
	Women	Men		Women	Men	
Number	910	1163		911	1162	
Demographics						
Age (years)	57.0 (9.2)	56.9 (10.0)	0.794	57.5 (9.7)	56.8 (9.7)	0.121
Body mass index (kg/m^2^)	29.9 (6.5)	29.6 (5.6)	0.229	30.4 (6.8)	29.2 (4.8)	<0.001
Atrial fibrillation	13 (1.4)	28 (2.4)	0.153	11 (1.2)	33 (2.8)	0.016
Cardiovascular risk factors						
Cigarette smoker^a^	458 (50.4)	632 (54.5)	0.067	452 (49.7)	643 (55.4)	0.011
Hypertension	303 (33.6)	380 (33.0)	0.795	304 (33.7)	408 (35.5)	0.428
Diabetes mellitus	83 (9.1)	138 (11.9)	0.053	77 (8.5)	146 (12.6)	0.003
Hypercholesterolaemia	502 (55.2)	679 (58.4)	0.154	513 (56.3)	716 (61.6)	0.017
Family history	432 (47.7)	397 (34.6)	<0.001	427 (47.5)	460 (39.9)	0.001
History of coronary heart disease	49 (5.4)	137 (11.8)	<0.001	49 (5.4)	137 (11.8)	<0.001
Medications						
Anti-platelet medication	401 (44.1)	583 (50.1)	0.021	399 (43.8)	610 (52.5)	<0.001
Statin	341 (37.5)	543 (46.7)	<0.001	338 (37.1)	564 (48.5)	<0.001
Beta-blockade	180 (19.8)	304 (26.1)	0.003	198 (21.7)	306 (26.3)	0.029
ACE-inhibitor/ARB	130 (14.3)	214 (18.4)	0.040	118 (13.0)	223 (19.2)	<0.001
Calcium channel blocker	84 (9.2)	110 (9.5)	0.919	80 (8.8)	103 (8.9)	0.527
Nitrates	241 (26.5)	349 (30.0)	0.193	219 (24.0)	351 (30.2)	0.004
Other antianginal therapy	31 (3.4)	44 (3.8)	0.736	29 (3.2)	49 (4.2)	0.267
Anginal symptoms^b^			0.118			<0.001
Typical	301 (33.1)	424 (36.5)		281 (30.9)	456 (39.2)	
Atypical	231 (25.4)	255 (22.0)		254 (27.9)	248 (21.3)	
Non-anginal	377 (41.5)	482 (41.5)		375 (41.2)	458 (39.4)	
Electrocardiogram						
Normal	783 (87.0)	952 (82.7)	0.009	789 (88.1)	968 (84.0)	0.010
Stress electrocardiogram			<0.001			<0.001
Performed	746 (82.3)	1007 (87.0)		756 (83.3)	1008 (87.1)	
Normal	477 (69.4)	612 (64.4)		491 (69.0)	616 (65.3)	
Inconclusive	129 (18.8)	154 (16.2)		139 (19.5)	146 (15.5)	
Abnormal^‡^	81 (11.8)	185 (19.5)		82 (11.5)	182 (19.3)	
Further investigations						
Stress imaging						
Radionuclide	129 (14.2)	84 (7.2)	<0.001	115 (12.6)	61 (5.2)	<0.001
Other	7 (0.8)	6 (0.5)	0.719	6 (0.7)	9 (0.8)	0.504
Invasive coronary angiography	76 (8.4)	184 (15.8)	<0.001	59 (6.5)	196 (16.9)	<0.001
Baseline diagnosis of angina due to CHD			<0.001			<0.001
No	103 (11.3)	163 (14.0)		110 (12.1)	157 (13.5)	
Unlikely	524 (57.6)	537 (46.3)		525 (57.7)	538 (46.3)	
Probable	239 (26.3)	363 (31.3)		240 (26.4)	362 (31.2)	
Yes	43 (4.7)	98 (8.4)		35 (3.8)	105 (9.0)	
Baseline diagnosis of CHD			<0.001			<0.001
No	49 (5.4)	83 (7.1)		58 (6.4)	80 (6.9)	
Unlikely	487 (53.6)	495 (42.6)		475 (52.2)	477 (41.0)	
Possible	314 (34.5)	420 (36.2)		328 (36.0)	444 (38.2)	
Yes	59 (6.5)	163 (14.0)		49 (5.4)	161 (13.9)	

Values are expressed as *n* (%) or mean ± standard deviation. Missing data (standard care alone, standard care + CTCA): atrial fibrillation *n* = 4 (3, 1); prior coronary heart disease *n* = 4 (3, 1); smoking habit *n* = 7 (5, 2); hypertension *n* = 41 (20, 21); hypercholesterolaemia *n* = 4 (3, 1); family history *n* = 43 (21, 22); angina symptoms *n* = 4 (3, 1); concomitant therapies *n* = 4 for all (3, 1 for all); resting electrocardiogram *n* = 46 (22, 24); exercise electrocardiogram *n* = 18 (10, 8); exercise electrocardiogram outcome *n* = 234 (121, 113); further investigations *n* = 6 (4, 2); stress imaging *n* = 4 (3, 1); coronary angiography *n* = 4 (3, 1); and baseline diagnosis *n* = 4 (3, 1).

ACE, angiotensin-converting enzyme; ARB, angiotensin receptor blocker; CHD, coronary heart disease.

a Current/ex-smokers.

b National Institute for Health and Care Excellence criteria.

### Findings on computed tomography coronary angiography

Of 2073 participants [*n* = 911 (44%) women] randomized to CTCA-guided management, 1778 participants underwent CTCA (*Table 2*). Eighty-one percent of women had a low coronary calcium score (<100 AU), vs. 53% of men (*P* < 0.001). On CTCA, the proportion of women with normal coronary arteries was two-fold higher than in men, whereas 105 (11.5%) of 911 women had obstructive CHD, which was nearly three-fold lower than in men [*n* = 347 (29.9%) of 1162, *P* < 0.001]. The proportions of men with single-, two-, and three-vessel obstructive CHD were two to five-fold higher than in women (*Table 2* and *Take home figure*).

**Take home figure:**
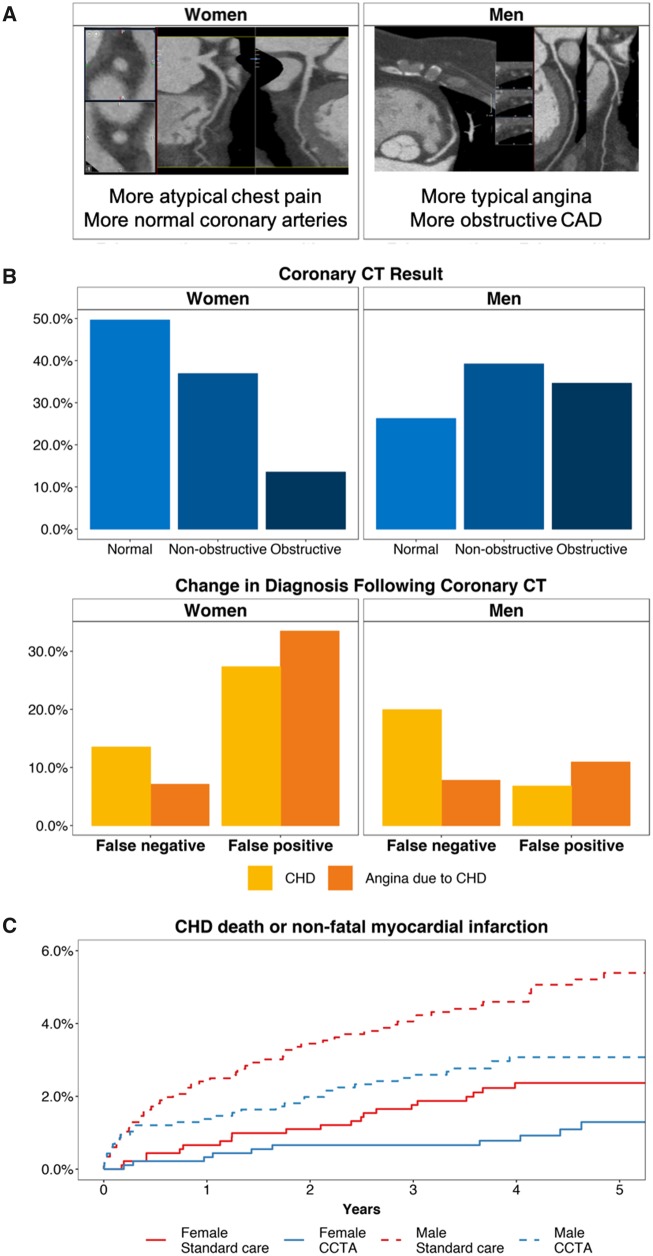
Women were more likely than men to have (*A*) atypical chest pain and normal coronary arteries leading to (*B*) greater false-positive diagnoses of coronary heart disease and angina due to coronary heart disease. Overall, women had low rates of clinical events (coronary heart disease death or non-fatal myocardial infarction) at 5 years but derived a similar prognostic benefit from computed tomography coronary angiography as men. (*C*) Cumulative event curves for the principal long-term clinical endpoint in those assigned to standard care plus computed tomography coronary angiography (blue) and standard care alone (red) amongst women (solid lines) and men (dashed lines).

**Table 2:** Findings disclosed by computed tomography coronary angiography in men and women

	Women (*N* = 911)	Men (*N* = 1162)	*P*-value
Coronary calcium score	*N* = 787	*N* = 1006	<0.001
Low (<100 AU)	638 (81.1)	529 (52.6)	
Medium (100–400 AU)	94 (11.9)	210 (20.9)	
High (>400 AU)	55 (7.0)	267 (26.5)	
Computed tomography coronary angiography	*N* = 774	*N* = 997	<0.001
Normal	384 (49.6)	263 (26.3)	
Non-obstructive CHD			
Mild (<50%)	172 (22.2)	200 (20.0)	
Moderate (50–70%)	113 (14.6)	187 (18.8)	
Obstructive CHD			
One-vessel	60 (7.8)	147 (14.7)	
Two-vessel	31 (4.0)	97 (9.7)	
Three-vessel	14 (1.8)	103 (10.3)	

Values are expressed as *n* (%).

AU, Agatston Units; CHD, coronary heart disease.

### Baseline and 6-week diagnoses of coronary heart disease and angina due to CHD

Overall, CTCA resulted in more frequent diagnostic changes in women than men (absolute risk difference 5.68, 95% CI: 2.71–8.65, *P*-interaction < 0.001). Because standard care-guided management also resulted in more frequent changes in the diagnosis of CHD amongst women than men, the relative benefits of CTCA were similar [relative risk 14.6, 95% CI 8.6–27.5 (women) vs. 16.9, 95% CI 9.2–35.6 (men), *Table 3*]. However, there were important differences in the direction of this change in diagnosis. CTCA-guided management was more likely to identify a false-positive baseline CHD diagnosis amongst women [103 of 377 (27.3%) (CTCA) vs. 9 of 373 (2.4%) (standard care), number needed to scan 4.0, 95% CI 3.4–5.0] compared with men [41 of 605 (6.8%) (CTCA) vs. 5 of 583 (0.9%) (standard care), number needed to scan 16.9, 95% CI 12.4–26.7] (Supplementary material online, *Table S2*, *Take home figure*). Conversely, the proportion of false-negative baseline diagnoses was similar for women [72 of 533 (13.5%) (CTCA) vs. 3 of 536 (0.5%) (standard care), number needed to scan 7.7, 95% CI 6.3–10.0] compared with men [111 of 557 (19.9%) (CTCA) vs. 3 of 578 (0.5%) (standard care), number needed to scan 5.2, 95% CI 4.4–6.3].

**Table 3:** Change in diagnosis of coronary heart disease and angina

Change in diagnosis of CHD			
Standard care, *N* (%)	No change	Change	
Female	898	12	
Male	1154	9	
CTCA, *N* (%)			
Female	736	175	
Male	1010	152	
	Female	Male	Interaction
Odds ratio	17.8 (10.3–34.0)	19.3 (10.4–41.0)	1.1 (*P* = 0.860)
Absolute risk change	17.9%	12.3%	*P* < 0.001
Difference in absolute risk	5.7 (2.7–8.7)		
Change in diagnosis of angina due to CHD			
Standard care, *N* (%)	No change	Change	
Female	900	10	
Male	1154	9	
CTCA, *N* (%)			
Female	774	137	
Male	1057	105	
	Female	Male	Interaction
Odds ratio	15.9 (8.8–32.6)	12.7 (6.8–27.2)	0.8 (*P* = 0.642)
Absolute risk change	13.9%	8.3%	*P* < 0.001
Difference in absolute risk	5.6 (2.2–8.8)		

Similarly, regarding the classification of angina due to CHD, CTCA changed the diagnosis in 54 (7.8%) of 694 men and 45 (7.1%) of 634 women thought not to have CHD and excluded the diagnosis in 51 (10.9%) of 467 men and 92 (33.7%) of 273 women (*Take home figure*). As before, CTCA changed the diagnosis of angina due to CHD more frequently in women compared with men (absolute risk difference 5.66, 95% 2.72–8.65, *P*-interaction = 0.007).

### Changes in investigations and treatment at 6 weeks

There were no differences in invasive coronary angiography or coronary revascularization rates between standard care and CTCA-guided care. CTCA-guided management resulted in cancellation of tests (myocardial perfusion imaging and stress echocardiography) with an absolute risk difference of 4.45 (95% CI: 2.25–6.65); *P* < 0.001) and changes in antianginal therapy [absolute risk difference: 4.5 (95% CI: 1.9–7.2), *P* < 0.001]. CTCA-guided management resulted in similar rates of changes to preventative therapy (*Table 4*).

**Table 4:** Changes in investigations and treatments at 6 weeks

Preventative medications—change			
Standard care, *N* (%)	No change	Change	
Female	872	38	
Male	1111	52	
CTCA, *N* (%)			
Female	749	162	
Male	955	207	
	Female	Male	Interaction
Odds ratio	5.0 (3.5–7.3)	4.6 (3.4–6.4)	*P* = 0.779
Absolute risk change	13.6%	13.3%	
Difference in absolute risk reduction	0.3 (−3.5 to 4.0)		*P* = 0.890
Antianginal medications—change			
Standard care, *N* (%)	No change	Change	
Female	902	8	
Male	1155	8	
CTCA, *N* (%)			
Female	802	109	
Male	1078	84	
	Female	Male	Interaction
Odds ratio	15.3 (7.9–34.4)	11.2 (5.8–25.3)	*P* = 0.556
Absolute risk change	11.1%	6.5%	
Difference in absolute risk reduction	4.5 (1.9–7.2)		*P* < 0.001
Stress imaging investigations—change			
Standard care, *N* (%)	No change	Change	
Female	906	4	
Male	1161	2	
CTCA, *N* (%)			
Female	832	79	
Male	1116	46	
	Female	Male	Interaction
Odds ratio	21.5 (8.9–70.7)	23.9 (7.4–146.8)	*P* = 0.904
Relative risk	19.7 (7.3–53.6)	23.9 (5.8–98.8)	
Absolute risk change	8.2%	3.8%	
Difference in absolute risk reduction	4.5 (2.3–6.7)		*P* < 0.001

CTCA, computed tomography coronary angiography.

### Angina

There were no sex differences in physical limitation, angina stability, frequency, satisfaction with treatment, and quality of life, as assessed using the Seattle Angina Questionnaire, at 6 weeks and 6 months, when compared with baseline observations (*Table 1*).

### Clinical endpoints

After a median of 4.8-year follow-up, women had a lower composite endpoint rate of death due to CHD or MI or death due to CHD, MI, or stroke than men (*Take home figure*). Crude differences in health outcomes and adjusted hazard ratios were observed between women and men for CTCA-guided management vs. standard care (*Table 5*).

**Table 5:** Clinical outcomes by sex and treatment group after a median of 4.8 years

	Standard care vs. CTCA						Interaction *P*-value^a^
	Women			Men			
	*N* (CTCA)	*N* (standard care)	HR (95% CI)	*N* (CTCA)	*N* (standard care)	HR (95% CI)	
CHD death or myocardial infarction	11 (1.2)	22 (2.4)	0.50 (0.24–1.04)	37 (3.2)	59 (5.1)	0.63 (0.42–0.95)	0.572
CHD death myocardial infarction or stroke	19 (2.1)	26 (2.9)	0.72 (0.40–1.30)	44 (3.8)	71 (6.1)	0.63 (0.43–0.91)	0.686
Cardiovascular events							
Myocardial infarction	11 (1.2)	21 (2.3)	0.53 (0.25–1.10)	33 (2.8)	52 (4.5)	0.64 (0.41–0.99)	0.638
Stroke	8 (0.9)	5 (0.5)	1.57 (0.50–4.89)	7 (0.6)	15 (1.3)	0.47 (0.19–1.15)	0.099
Death							
CHD	0 (0.0)	1 (0.1)	—	4 (0.3)	8 (0.7)	0.51 (0.15–1.71)	—
Cardiovascular	1 (0.1)	1 (0.1)	1.42 (0.08–24.38)	4 (0.3)	11 (0.9)	0.38 (0.12–1.20)	0.532
Non-cardiovascular	12 (1.3)	8 (0.9)	1.45 (0.59–3.56)	26 (2.2)	23 (2.0)	1.17 (0.67–2.06)	0.701
All-cause	13 (1.4)	9 (1.0)	1.42 (0.60–3.33)	30 (2.6)	34 (2.9)	0.92 (0.56–1.50)	0.403
Procedures							
Coronary angiography	144 (15.8)	159 (17.5)	0.86 (0.69–1.09)	347 (29.9)	343 (29.5)	1.06 (0.91–1.22)	0.171
Coronary revascularization	53 (5.8)	45 (4.9)	1.15 (0.77–1.72)	226 (19.4)	222 (19.1)	1.05 (0.87–1.27)	0.652

Hazard ratios (HRs) were determined with Cox regression models adjusted for centre and minimization variables (age, body mass index, diabetes mellitus, prior coronary heart disease, and atrial fibrillation).

a *P*-value for the interaction between sex and allocated treatment.

## Discussion

We have undertaken an analysis by sex of the main findings in the SCOT-HEART trial. Compared with men, women had differences in the typicality of their anginal symptoms, a higher likelihood of having normal coronary arteries, and more frequent diagnostic and therapeutic changes with CTCA-guided management. In particular, CTCA modified apparent over-diagnosis and treatment of women who had been incorrectly diagnosed with CHD and angina due to CHD. Both women and men appear to benefit equally from the addition of CTCA to standard care with no evidence of an interaction between sex and health outcomes identified.

In line with prior reports,^17–19^ women reported less typical anginal symptoms making clinical assessment more challenging. This diagnostic uncertainty, and lower prevalence of obstructive CHD, led to an over-diagnosis of CHD and angina. Exercise electrocardiography testing has limited sensitivity and specificity for the presence of coronary artery disease, especially in women^17^ which may have contributed to misdiagnosis. Indeed, women were more likely to have downstream non-invasive stress testing cancelled, and antianginal therapies reduced following reclassification by CTCA. On the other hand, ischaemia and no obstructive coronary artery disease (INOCA) caused by small vessel disease more commonly affects women.^20^ We suggest that CTCA-guided diagnosis and management is helpful in women for the diagnosis of angina due to CHD, but less so for INOCA. These findings extend the sex subanalysis of the CRESCENT (Calcium Imaging and Selective CT Angiography in Comparison to Functional Testing for Suspected Coronary Artery Disease) trial.^21^

When compared with exercise electrocardiography testing, CTCA-guided therapy impacted on false-positive classifications without affecting false-negative classifications in women. This is in contrast to data published from the PROspective Multicentre Imaging Study for Evaluation of chest pain (PROMISE) trial, where statin therapy was lower in women than in men and women were less likely to be referred for coronary angiography^19^. In PROMISE, 88% of participants had chest pain (72%) or an anginal equivalent (16%), whilst 10% had typical angina compared with SCOT-HEART where 100% had chest pain, 35% had typical angina and 9% had known CHD. The standard of care was exercise electrocardiography and the results of a trial involving different functional tests as standard of care might be different.

In our study, patients were recruited from cardiology clinics rather than general outpatient clinics. In SCOT-HEART, clinicians were free to request other non-invasive stress imaging at their discretion and indeed 10% of additional testing was requested, mostly radionuclide scintigraphy. In contrast, in the functional testing arm of PROMISE, radionuclide scintigraphy predominated (67%) with stress echocardiography and electrocardiography accounting for the remainder. We found that normal coronary arteries were two-fold more common in women whereas obstructive CHD was three-fold more common in men, similar to Pagidipati *et al*.^19^ This has important therapeutic implications for coronary revascularization rates and use of medical therapy across genders.^10^ Symptoms and quality of life improve when CTCA-guided management discloses normal coronary arteries^22^ and this favourable outcome associates with female sex. Typical anginal symptoms and obstructive CHD were less common in women, reflecting aetiological differences, and manifest by lower rates of coronary revascularization.^17^^,^^18^ These findings extend those of PROMISE.^23^

Women are more likely to experience angina due to small vessel disease,^20^ whereas obstructive CHD is more common in men. The CorMicA trial recently provided evidence that in patients with angina and no obstructive CHD, stratified medicine including adjunctive tests of small vessel function leads to improvements in angina and quality of life.^20^ The prevalence and clinical significance of small vessel disease in patients with chest pain and normal coronary arteries or non-obstructive CHD is being prospectively assessed in in the Coronary Microvascular Function and CT Coronary Angiography (CorCTCA) trial.^24^

There are sex differences in prognosis following a diagnosis of stable angina, which is notably worse in younger women than in men.^17^ These differences may be explained by under-use of relevant tests and treatments.^17^ In our study, women had lower crude rates of adverse cardiovascular events when compared with men, and a lower rate of MI in both treatment arms. The magnitude and direction of the benefits of CTCA on fatal and non-fatal MI were similar between the sexes. There were no differences in the longer-term in coronary angiography and revascularization rates between groups for both women and men. There was a small numerically higher rate of non-cardiac death and stroke events in women in the CT group vs. the standard care group. The number of events was very low and difficult to interpret.

The interaction tests for sex, treatment group allocation, and health outcomes were not statistically significant (i.e. the null hypothesis was not rejected). Thus, the benefits of CTCA-guided management on health outcomes appear to be similar in women and men. This finding contrasts with the PROMISE^25^ where women appeared to gain more prognostic benefit than men from a CTCA-guided strategy. A gender-specific *post hoc* analysis from DISCHARGE (Diagnostic Imaging Strategies for Patients With Stable Chest Pain and Intermediate Risk of Coronary Artery Disease, ClinicalTrials.gov identifier: NCT02400229)^26^ would extend whether women benefit from CTCA-guided strategy vs. invasive coronary angiography in the investigation of CHD.

### Limitations

There are a number of limitations associated with this study. First, this was a *post hoc* analysis of an open-label trial and gender was not randomized. Second, this study was not designed or powered for this secondary analysis, and our findings are exploratory. Third, the small numbers of changes in the standard care arm resulted in a large variability in the relative changes that it was not possible to draw any firm conclusions from the logistic regression analyses. However, absolute differences allow for different proportions of changes in the diagnosis and establishes the gender differences we report. Finally, information on microvascular dysfunction as an alternative cause of angina was not available. Further studies are on-going.^24^

## Conclusions

Women are less likely to have typical symptoms or obstructive CHD but are more likely to be over-diagnosed. CTCA is useful in reducing over-diagnosis and medication in women and identifies unrecognized CHD equally in both sexes with similar prognostic benefits. More research is needed to determine the causes of, and treatments for, angina in women and men with angiographically normal coronary arteries.

## Supplementary Material

ehz903_Online_Supplementary
