# Supplementary material for: Sex associations and computed tomography coronary angiography-guided management in patients with stable chest pain
Source: Eur Heart J. 2019 Dec 28;41(13):1337–45. doi: 10.1093/eurheartj/ehz903 (PMC7109601; doi:10.1093/eurheartj/ehz903)
Supplement: ehz903_Online_Supplementary [file ehz903_online_supplementary.doc]

# ONLINE SUPPLEMENT

# Sex-associations and CT-coronary angiography-guided management in patients with stable chest pain.

## Methods

### Study criteria

All patients underwent routine clinic assessment including, if deemed appropriate, symptom-limited exercise electrocardiography with the standard Bruce protocol. Symptoms (typical, atypical, or non-anginal chest pain according to the National Institute for Health and Care Excellence, diagnosis, investigations, and treatment strategy were documented at the

end of the clinic attendance, before randomisation. This included categorising (no, unlikely, probable, or yes) the likelihood of the diagnosis of coronary heart disease and

angina due to coronary heart disease and documentation of the need for additional stress imaging, such as stress echocardiography, and radionuclide or magnetic resonance

myocardial perfusion imaging, and invasive coronary angiography.

### Statistical analyses

Data are reported as means and standard deviations, medians and interquartile ranges, and hazard ratios with 95% confidence intervals, as appropriate. No adjustment for multiplicity was undertaken, so intervals and p-values should not be used to infer definitive treatment effects.

### Role of the funding source

The funder had no role in the trial conduct including data collection, analysis, interpretation, writing of the manuscript, and the decision to submit. The data were analysed by PDA. The Trial Steering Committee and the Chief Investigator were responsible for the decision to submit the manuscript. Toshiba, Siemens, and Philips had no role in the trial conduct.

### Supplementary Table 1. Changes from baseline in Seattle Angina Questionnaire after 6 weeks and 6 months in men and women randomised to standard care plus computed tomography coronary angiography and standard care alone.

|  | Standard Care + CTCA |  | Standard Care |  | Difference  [95% Confidence Intervals] | P value  (for difference) | Difference  [95% Confidence Intervals] | P value  (for difference) |
| --- | --- | --- | --- | --- | --- | --- | --- | --- |
|  | Women | Men | Women | Men | Women | Women | Men | Men |
| Change at 6 weeks |  |  |  |  |  |  |  |  |
| Physical Limitation | 0.08 | -0.54 | 0.59 | -0.47 | -0.51 (-0.55, 1.58) | 0.345 | -0.07 (-0.86, 1.00) | 0.882 |
| Angina Stability | 12.07 | 14.05 | 12.17 | 11.63 | -0.10 (-2.76, 2.95) | 0.948 | 2.42 (-5.07, 0.23) | 0.073 |
| Angina Frequency | 9.4 | 8.92 | 9.77 | 8.4 | -0.37 (-1.61, 2.36) | 0.712 | 0.51 (-2.25, 1.22) | 0.562 |
| Treatment Satisfaction | -5.3 | -5.88 | -5.08 | -5.5 | -0.22 (-1.15, 1.60) | 0.753 | -0.38 (-0.88, 1.64) | 0.553 |
| Quality of Life | 8.23 | 6.11 | 8.4 | 6.84 | -0.17 (-1.59, 1.93) | 0.851 | -0.72 (-0.93, 2.37) | 0.391 |
| Change at 6 months |  |  |  |  |  |  |  |  |
| Physical Limitation | 0.46 | 0.92 | 1.14 | 1.4 | -0.68 (-0.44, 1.80) | 0.233 | -0.47 (-0.51, 1.45) | 0.342 |
| Angina Stability | 10.54 | 8.63 | 7.97 | 8.53 | 2.57 (-5.21, 0.07) | 0.056 | 0.09 (-2.50, 2.31) | 0.939 |
| Angina Frequency | 12.95 | 13.49 | 12.89 | 12.93 | 0.06 (-2.05, 1.93) | 0.951 | 0.56 (-2.31, 1.19) | 0.529 |
| Treatment Satisfaction | -3.34 | -3.8 | -2.75 | -2.96 | -0.58 (-0.68, 1.85) | 0.175 | -0.83 (-0.37, 2.03) | 0.366 |
| Quality of Life | 11.52 | 10.75 | 12.31 | 12.44 | -0.79 (-1.06, 2.64) | 0.058 | -1.69 (-0.06, 3.44) | 0.402 |

Confidence intervals and p-values determined from subgroup t-tests and not adjusted for multiple comparisons.

### Supplementary Table 2 Baseline and 6-week diagnosis of coronary heart disease in men

| Diagnosis of CHD |  |  | 6-week diagnosis |  |
| --- | --- | --- | --- | --- |
| CTCA |  | Negative (Unlikely/No) | Positive (Yes/Probable) | Total |
| Baseline diagnosis | Negative (Unlikely/No) | 446 (38.4%) | 111 (9.6%) | 557 (47.9%) |
|  | Positive (Yes/Probable) | 41 (3.5%) | 564 (48.5%) | 605 (52.1%) |
|  | Total | 487 (41.9%) | 675 (58.1%) | 1162 (100.0%) |
|  |  |  |  |  |
| Standard Care |  | Negative (Unlikely/No) | Positive (Yes/Probable) | Total |
| Baseline diagnosis | Negative (Unlikely/No) | 574 (49.4%) | 4 (0.3%) | 578 (49.8%) |
|  | Positive (Yes/Probable) | 5 (0.4%) | 578 (49.8%) | 583 (50.2%) |
|  | Total | 579 (49.9%) | 582 (50.1%) | 1161 (100.0%) |

| Diagnosis of angina due to CHD |  |  | 6-week diagnosis |  |
| --- | --- | --- | --- | --- |
| CTCA |  | Negative (Unlikely/No) | Positive (Yes/Probable) | Total |
| Baseline diagnosis | Negative (Unlikely/No) | 640 (55.1%) | 54 (4.7%) | 694 (59.8%) |
|  | Positive (Yes/Probable) | 51 (4.4%) | 416 (35.8%) | 467 (40.2%) |
|  | Total | 691 (59.5%) | 470 (40.5%) | 1161 (100.0%) |
|  |  |  |  |  |
| Standard Care |  | Negative (Unlikely/No) | Positive (Yes/Probable) | Total |
| Baseline diagnosis | Negative (Unlikely/No) | 696 (59.9%) | 4 (0.3%) | 700 (60.3%) |
|  | Positive (Yes/Probable) | 5 (0.4%) | 456 (39.3%) | 461 (39.7%) |
|  | Total | 701 (60.4%) | 460 (39.6%) | 1161 (100.0%) |

### Supplementary Table 2. ctd. Baseline and 6-week diagnosis of coronary heart disease in women.

| Diagnosis of CHD |  |  | 6-week diagnosis |  |
| --- | --- | --- | --- | --- |
| CTCA |  | Negative (Unlikely/No) | Positive (Yes/Probable) | Total |
| Baseline diagnosis | Negative (Unlikely/No) | 461 (50.7%) | 72 (7.9%) | 533 (58.6%) |
|  | Positive (Yes/Probable) | 103 (11.3%) | 274 (30.1%) | 377 (41.4%) |
|  | Total | 564 (62.0%) | 346 (38.0%) | 910 (100.0%) |
|  |  |  |  |  |
| Standard Care |  | Negative (Unlikely/No) | Positive (Yes/Probable) | Total |
| Baseline diagnosis | Negative (Unlikely/No) | 533 (58.6%) | 3 (0.3%) | 536 (59.0%) |
|  | Positive (Yes/Probable) | 9 (1.0%) | 364 (40.0%) | 373 (41.0%) |
|  | Total | 542 (59.6%) | 367 (40.4%) | 909 (100.0%) |

| Diagnosis of angina due to CHD |  |  | 6-week diagnosis |  |
| --- | --- | --- | --- | --- |
| CTCA |  | Negative (Unlikely/No) | Positive (Yes/Probable) | Total |
| Baseline diagnosis | Negative (Unlikely/No) | 589 (64.9%) | 45 (5.0%) | 634 (69.9%) |
|  | Positive (Yes/Probable) | 92 (10.1%) | 181 (20.0%) | 273 (30.1%) |
|  | Total | 681 (75.1%) | 226 (24.9%) | 907 (100.0%) |
|  |  |  |  |  |
| Standard Care |  | Negative (Unlikely/No) | Positive (Yes/Probable) | Total |
| Baseline diagnosis | Negative (Unlikely/No) | 625 (68.8%) | 2 (0.2%) | 627 (69.0%) |
|  | Positive (Yes/Probable) | 8 (0.9%) | 274 (30.1%) | 282 (31.0%) |
|  | Total | 633 (69.6%) | 276 (30.4%) | 909 (100.0%) |

n (%); Clinicians were asked to diagnose both (i) coronary heart disease, and (ii) angina due to coronary heart disease, in the light of all the information available to them. They were asked to categorise this according to the level of confidence in their diagnosis (yes/probable, unlikely/no) both at baseline and at 6 weeks after the result of the CTCA (standard care and CTCA) and ASSIGN score (standard care). Frequency of the diagnosis was compared between yes/probable and unlikely/no. Complete agreement at baseline and 6 weeks shown by the grey shaded boxes.
